# Construction of an MUC-1 promoter driven, conditionally replicating adenovirus that expresses the sodium iodide symporter for gene therapy of breast cancer

**DOI:** 10.1186/bcr2342

**Published:** 2009-07-27

**Authors:** Miguel A Trujillo, Michael J Oneal, Julia Davydova, Elizabeth Bergert, Masato Yamamoto, John C Morris

**Affiliations:** 1 Department of Internal Medicine, Division of Endocrinology, Diabetes, Metabolism, Nutrition, Mayo Clinic Rochester, 200 First Street SW, Rochester, MN 55905, USA; 2 Department of Molecular Medicine, Mayo Clinic Rochester, 200 First Street SW, Rochester, MN 55905, USA; 3 Department of Surgery, University of Minnesota, MMC195, 420 Delaware St., SE Minneapolis, MN 55455, USA

## Abstract

**Introduction:**

The sodium iodide symporter (NIS) directs the uptake and concentration of iodide in thyroid cells. This in turn allows radioiodine imaging and therapy for thyroid cancer. To extend the use of NIS-mediated radioiodine therapy to other types of cancer, we successfully transferred and expressed the sodium-iodide symporter (*NIS*) gene in prostate, colon, and breast cancer cells both *in vivo *and *in vitro *by using non-replicating adenoviral vectors.

**Methods:**

To improve virotherapy efficiency, we developed a conditionally replicating adenovirus (CRAd) in which the transcriptional cassette RSV promoter-human NIScDNA-bGH polyA was also inserted at the E3 region. The *E1a *gene is driven by the tumor-specific promoter MUC-1 in the CRAd Ad5AMUCH_RSV-NIS.

**Results:**

*In vitro *infection of the MUC-1-positive breast cell line T47D resulted in virus replication, cytolysis, and release of infective viral particles. Conversely, the MUC-1-negative breast cancer cell line MDA-MB-231 was refractory to the viral cytopathic effect and did not support viral replication. The data indicate that Ad5AMUCH_RSV-NIS activity is stringently restricted to MUC-1-positive cancer cells. Radioiodine uptake was readily measurable in T47 cells infected with Ad5AMUCH_RSV-NIS 24 hours after infection, thus confirming NIS expression before viral-induced cell death.

**Conclusions:**

This construct may allow multimodal therapy, combining virotherapy with radioiodine therapy to be developed as a novel treatment for breast and other MUC1-overexpressing cancers.

## Introduction

The American Cancer Society's most recent estimates for breast cancer in the United States for 2009 are as follows: 192,370 new cases of invasive breast cancer and 40,170 deaths of breast cancer. Breast cancer is the most common cancer among women in the United States, other than skin cancer. It is the second leading cause of cancer death in women, after lung cancer [1]. Breast cancer is particularly difficult to treat when it metastasizes and becomes resistant to antiestrogen therapies [2].

The sodium iodide symporter (NIS) is a transmembrane glycoprotein that mediates uptake of iodide into cells, especially thyroid follicular cells [3,4]. The presence of NIS on the basolateral membrane of thyroid cells has been exploited for many years for diagnostic imaging purposes, as well as for ablative therapy of differentiated thyroid cancer by using radioactive iodide (^131^I). This noninvasive therapy has proven to be a safe and effective treatment for thyroid cancer, even in advanced, metastatic disease [5,6]. To extend the use of NIS-mediated radioiodine therapy to other types of cancer, we successfully transferred and expressed the sodium-iodide symporter (*NIS*) gene in prostate, colon, and breast cancer cells, both *in vivo *and *in vitro*, by using adenoviral vectors. Our experience with adenovirus-mediated *NIS *transfer and radioiodine therapy was confirmed in a large animal model and has culminated in the opening of a phase I trial for prostate cancer that is currently in progress [7-12].

MUC-1 is a highly glycosylated transmembrane mucin. Although MUC-1 is expressed at very low levels in normal tissues; it is overexpressed by most carcinomas, including breast cancers [13]. Enhanced expression of *MUC-1 *is regulated mostly at the level of transcription [14]. The pronounced differential expression of MUC-1 in tumor versus normal tissues has been used in experimental developments of antitumor therapies, including MUC-1 vaccines and MUC-1 promoter-restricted antitumor-specific viruses [15-17].

All gene-therapy approaches depend on the ability to deliver therapeutic genes to target cells. However, the limited ability to transduce tumors efficiently with effective levels of therapeutic transgenes has been identified as the fundamental barrier to effective cancer gene therapy [18,19]. To address this issue, conditionally replicating viruses, including adenovirus, have been constructed, and their efficacy, evaluated [20-22].

Our approach to the current problems associated with virotherapy/gene therapy has been the development of tumor-specific, conditionally replicating adenoviral vectors that also harbor the *NIS *gene. We report here the development of a conditionally replicating adenovirus (CRAd) in which the E1a gene is driven by the tumor-specific promoter MUC-1. In addition, the transcriptional cassette RSV promoter-hNIScDNA-bGH polyA was inserted at the E3 region. Our results suggest that this CRAd may represent a novel approach to breast cancer gene therapy.

## Materials and methods

### Cell culture

The breast cancer cell lines T47D and MDA-MB-231 were used to examine CRAd Ad5AMUCH_RSV-NIS specificity. Adenovirus infection was performed for 4 hours in serum-free media. Cells were then washed once with PBS and replenished with fresh culture media. The human embryonic kidney cell line stably expressing E1A (HEK 293) was obtained from Cell Biolabs, Inc., San Diego, CA. Cells were cultured as described [8,23].

### CRAd construct and cell lines

The E1A gene flanked by a 5' blunt end, and an *Eco*RI 3' end was isolated from plasmid pCD512_F2 (a gift of Dr. Richard Vile, Mayo Clinic) and cloned into the Promega vector pGL3-Basic to yield pGL3E1. The MUC-1 promoter (a gift of Dr. Sandra Gendler, Mayo Clinic Arizona) was PCR amplified by using the forward primer AAAAAGGTACCGGACCCTAGGGTTCATCGGAG and the reverse primer AAAAAAGATCTGATTCAGGCAGGCGCTGGCTGC from the plasmid pcDNA3 [8]. The resulting PCR fragment 0.726 Kb spanning nt2188 to nt2914 was cloned into pGL3E1 to yield pGL3MUCH. The MUC-1 promoter-E1A-SV40 PolyA fragment was excised from pGL3MUCH by digestion with *Eco*RI/*Kpn*I and cloned into the shuttle vector pVQA-PB-NIS [23] restricted by *Kpn*I/*Eco*RI and dephosphorylated with CIP to generated pVQAMUCH. The human *NIS *cDNA under Rous Sarcoma Virus LTR promoter control was subcloned into an Ad5 genomic vector containing an E3-deleted version (ViraQuest, North Liberty, IA, USA). The pVQAMUCH shuttle vector and the *NIS*-containing Ad5 genomic plasmid were recombined to yield the CRAd Ad5AMUCH_RSV-NIS. Plaques were grown, purified, and characterized by ViraQuest as previously described [23]. The replication-incompetent Muc promoter-driven luciferase expression vector, AdEasyMucLuc, was constructed through homologous recombination in *Escherichia coli *by using the AdEasy system [24]. This vector has the transgene cassette in the E1 deleted region of an adenoviral vector backbone. The vector was constructed as follows: the MUC_1 promoter was derived from a plasmid pGL3MUCH and cloned into plasmid pShuttleGL3B [25] by using *Kpn*I and *Hin*dIII sites. The resultant plasmid, pShuttleMuc_GL3B, was linearized with *Pme*I digestion and subsequently co-transformed into *E. coli *BJ5183 with the pAdEasy-1 Ad backbone plasmid. After selection of recombinants in these bacteria, the recombinant DNA was linearized with *Pac*I digestion and transfected into 293 cells to generate AdEasyMucLuc. The virus was propagated in 293 cells, dialyzed in phosphate-buffered saline (PBS) with 10% glycerol, and stored at -80°C. Titering was performed with a plaque-forming assay by using 911 cells and optical density-based measurement.

### MUC-1 flow cytometry

The 10^6 ^exponentially growing cells were washed with PBS containing 1 mmol/L EDTA and resuspended in PBS. Cells were incubated with 20 μg/ml mouse anti-human MUC-1 antibody (Cell Signaling Technology Inc. Beverly, MA, USA) 30 min at RT. Controls cells were processed equally, except that they were incubated with 20 μg/ml mouse anti-human (IgG_1_) TNP anti-isotype antibody control (BD Biosciences Pharmingen, SanDiego, CA, USA). Cells were then washed in PBS and incubated with FITC-conjugated goat anti-mouse IgG secondary antibody (BD Biosciences Pharmingen) (1:100) for 30 min at RT. Cells were then washed, resuspended in PBS, and immediately analyzed with a Becton Dickinson FACScan by using CELLQuest software.

### Protein isolation and Western blots

For all Western blots, the protein concentration was measured in triplicate, and two gels were run. The first was developed with silver staining by using the PlusOne Silver Staining Kit, Protein (GE Healthcare Bio-Sciences, Uppsala, Sweden) according to the manufacturer's instruction and quantitated by using the ImageJ software [26]. This gel served as loading control (not shown). The second gel was blotted onto nitrocellulose and Western blotted. E1a and hexon extracts were prepared from 4 × 10^6 ^cells grown on 100-mm tissue-culture plates and infected at MOI 20. At noted time points, cells were rinsed twice in HBSS before lysis in 1 ml of the complete lysis-M reagent with supplied protease inhibitors (Roche Applied Science, Indianapolis, IN, USA) on ice with shaking for 5 minutes and then quantified by using the DC protein assay (Bio-Rad Hercules, CA, USA). Membrane proteins extracts were prepared for NIS and CAR Western blotting as described [27]. Lysate aliquots, 1 μg of protein, were electrophoresed by using I a X cell II Blot Module (Invitrogen, Carlsbad, CA, USA) on 10% BIS-TRIS polyacrylamide gels under reducing conditions at 200 V for 1 hour. Proteins were then blotted to nitrocellulose membranes at 25 V for 1 hour and processed with the ECL advanced Western blot detection kit (GE Healthcare, Uppsala, Sweden) according to the manufacturer's instructions. E1A primary antibody (Santa Cruz Biotechnology, Santa Cruz, CA, USA) was diluted 1:20,000, and the anti-Ad5 hexon IgG (Santa Cruz Biotechnology), 1:100,000. Anti-CAR analyses were performed by running 5 μg of membrane protein or 1 μg of cell extract on a 10% Bis-Tris polyacrylamide gels under nonreducing conditions. Blocking steps were performed as described earlier. Blots were incubated with primary mouse Anti-CAR, clone RmcB (Upstate Cell Signaling Solutions Inc., Danvers, MA, USA). The antibody was diluted 1:10,000 in TBS-T with 0.1% blocking reagent for 90 minutes. NIS Western blots were performed as previously described [27].

### Cytopathic effect assays

The 1 × 10^6 ^cells were infected at MOI 1. At noted time points, the cells were photographed under a light microscope at 100× magnification.

### MTS assay

The 5 × 10^4 ^cells were seeded in 96-well tissue-culture plates and incubated at 37°C, 5% CO_2 _in triplicate. Twenty-four hours after plating, the cells were infected at the indicated MOI. The virus-containing medium was exchanged with fresh medium, and at each time point, 50 μl of CellTiter 96 Aqueous One Solution Cell Proliferation Assay (Promega Corporation, Madison, WI, USA) was applied and then incubated at 37°C, 5% CO_2 _for 2 hours. Absorbance was then read at 490 nm by using a plate spectrophotometer.

### Replication assay of the CRAd vector

The 4 × 10^6 ^cells were plated in 150-cm^2 ^culture flasks and infected at MOI 1 and MOI 0.1. Seventy-two hours after infection, virus was harvested from media and cells separately and purified by using the ViraBind Adenovirus Purification Kit (Cell Biolabs, San Diego, CA, USA). Virus titers were quantified by plaque assay on HEK 293 cells.

### ^125^I uptake

Uptake of ^125^I by virus-infected cells was measured as described [10,28]. In brief, 1 × 10^6 ^cells/well were plated on 36-well tissue-culture plates and infected at MOI 0, 0.5, 5, 10, 20, and 50. At indicated time points, iodide-uptake measurement was performed in triplicate in HBSS supplemented with 10 mmol/L HEPES, pH 7.3, 10 μmol/L NaI, and 1 × 10^5 ^cpm of ^125^I. Control wells were incubated in the same buffer plus 10 μmol/L KClO_4_. Plates were gently mixed and incubated at 37°C, 5% CO_2_, for 45 minutes. After incubation, buffers were aspirated and the cells gently washed once with 4°C 10 mmol/L HEPES, pH 7.3 buffer. Cells were lysed in 1 ml 1 N NaOH with shaking for 20 minutes at RT, and lysates were assayed for ^125^I on a gamma counter.

### Noninvasive imaging of xenografted tumors

All experimental protocols were reviewed and approved by the Mayo Clinic Institutional Animal Care and Use Committee (IACUC). Xenografts derived from the T47D cell line were established on each flank of 6-week-old athymic nude Foxn1nu mice (Harlan, Madison, WI, USA) by subcutaneous injection of 4 × 10^6 ^cells resuspended in 0.125 μl media and 0.125 μl of BD Matrigel basement membrane matrix (BD Biosciences, Bedford, MA). Mice were maintained on a low-iodine diet and T_4 _supplementation (5 mg/L) in their drinking water throughout the duration of the experimentation to maximize radioisotope uptake in the tumor and minimize uptake by the thyroid [8-10]. The mice were examined daily for tumor growth. Tumor volume was measured with calipers twice weekly and calculated by the formula:



When tumors reached 100 mm^3^, mice received one intratumoral (IT) injection of 10^11 ^vp of the AdAMUCH_RSV-NIS CRAd on the left flank; the right flank served as control. Two days later, an intraperitoneal dose of 0.5 mCi of ^123^I sodium iodide was given, and 1 hour later, radioiodine imaging was performed by using a γ-camera equipped with a low-energy high-resolution collimator (VG System; GE Healthcare, Milwaukee, WI). Regions of uptake were quantified and expressed as a fraction of the total amount of the applied radioiodine.

## Results

### Virus construction

**Figure 1:**
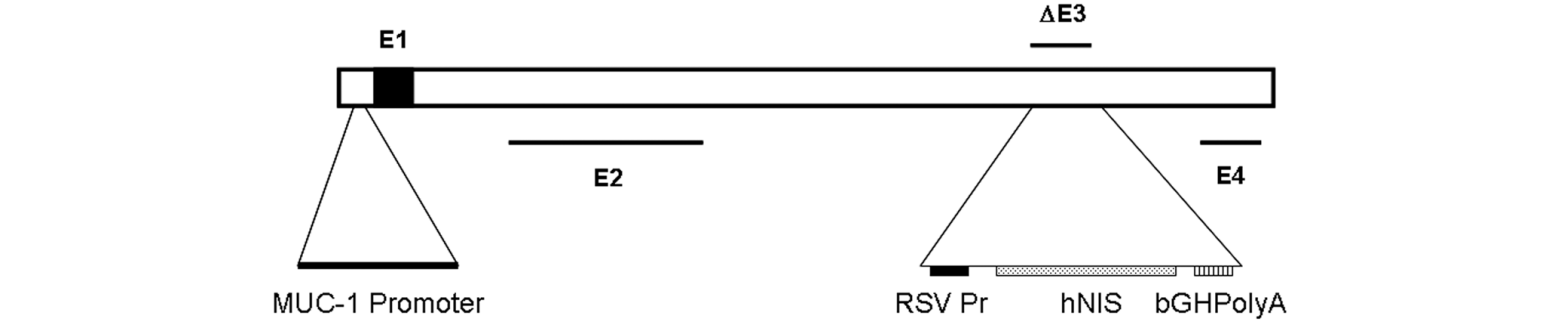
Structure of the Ad5AMUCH_RSV-NIS CRAd. The *E1A *gene is driven by the tumor-specific mucin-1 (MUC-1) promoter. The Rous sarcoma virus (RSV) promoter drives expression of the sodium iodide symporter (*NIS*) gene inserted at the E3 region.

The conditionally replicating adenoviruses (CRAd) Ad5AMUCH_RSV-NIS harbors the transcriptional cassette RSV promoter-human NIScDNA-bGH polyA inserted at the E3 region. In this CRAd, the *E1a *gene is driven by a 0.726-Kb version of the tumor-specific MUC-1 promoter spanning nt2188 to nt2914 [17] (Figure 1).

### MUC-1 and CAR expression

**Figure 2:**
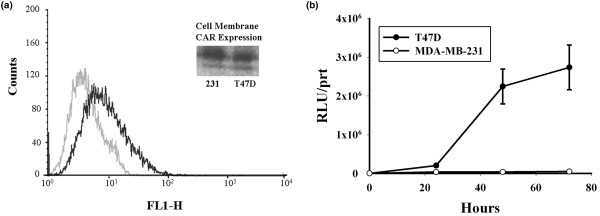
Mucin-1 (MUC-1) and coxsackievirus adenovirus receptor (CAR) expression. **(a) **Relative membrane MUC-1 expression in T47D (black line) and MDA-MB-231 cells (gray line) was measured with flow cytometry. Insert: Western blot with an anti-CAR receptor antibody of membrane proteins extracted from T47D and MDA-MB-231 cells. **(b) **MUC-1 promoter expression was monitored in T47D and MDA-MB-231 by measuring MUC-1-driven expression of luciferase activity after transduction with the nonreplicating virus AdEasyGL3B-Muc.

Two breast cancer cell lines, T47D and MDA-MB-231, were characterized for their expression of MUC-1. No difference in the flow-cytometry histogram was found between the isotype control and the MUC-1 antibody in MDA-MB-231, confirming that this cell line is MUC-1^- ^(data not shown). We then zeroed the FACScan by using this cell line and analyzed the T47D under this new setting. This analysis revealed that T47D is MUC-1 positive, whereas MDA-MB-231 is MUC-1 negative (Figure 2a). Moreover, when these two cell lines were infected with the nonreplicating virus AdEasyGL3B-Muc, in which the *Luc *gene is driven by the MUC-1 promoter, only the T47D cell line supported MUC-1-driven Luc activity, whereas MDA-MB-231 was completely negative (Figure 2b). To exclude the possibility that inability to support viral replication was due to lack of CAR-receptor expression, CAR-receptor expression was assayed with Western blot of membrane extracts prepared from the two cell lines. To control for protein loading, identical amounts of protein were loaded on a second gel that was silver stained for protein assessment (not shown). The insert in Figure 2a supports the notion that both cell lines readily express the CAR receptor at the cell plasma membrane. We conclude that T47D is MUC-I positive, whereas MDA-MB-231 is MUC-1 negative, and that both cell lines express CAR and thus are capable of being infected by wild-type capsid Ad5.

### Conditional replication, oncolysis, and spread of Ad5AMUCH_RSV-NIS

**Figure 3:**
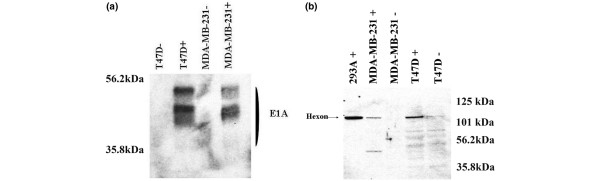
Viral proteins expression. Expression of E1A **(a) **was assayed in T47D and MDA-MB-23) cells. Cells were infected with Ad5AMUCH_RSV-NIS at multiplicity of infection (MOI) 20, and extracts were prepared at 24 hours after infection and compared with extracts from noninfected cells. **(b) **Hexon expression was assayed in MDA-MB-231 and T47D 48 hours after infection and compared with uninfected cells. HEK 293 cells infected with Ad5AMUCH_RSV-NIS were used as control.

We examined the specificity of Ad5AMUCH_RSV-NIS replication by using several methods. First, we examined the specificity of viral proteins expression. MUC-1-driven E1A and Hexon expression were examined with Western blotting. T47D and MDA-MB-2321 cells were infected with Ad5AMUCH_RSV-NIS at MOI 20. Figure 3 shows two major differences between the permissive T47D cell line and the nonpermissive MDA-MB-231. Only cells infected with Ad5AMUCH_RSV-NIS expressed viral proteins. Most important, a marked difference was noted in the quality and quantities of viral protein expressed in the permissive cell compared with the nonpermissive cell line (Figure 3a). We also examined hexon expression, a product of late protein synthesis and a very good indicator of viral assembly [29]. Here again, the amount of hexon protein produced 48 hours after infection in the permissive cell line T47D outperformed that produced in nonpermissive cell lines MDA-MB-231 (Figure 3b).

**Figure 4:**
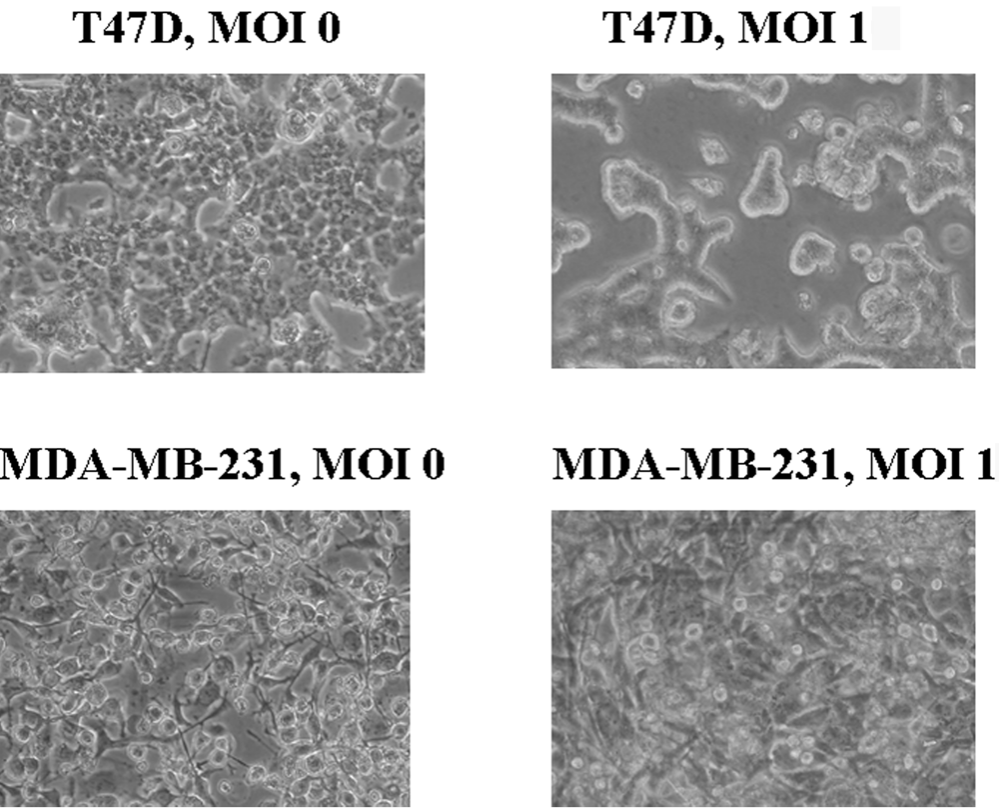
Cytopathic effect. Ad5PB_RSV-NIS-induced cytopathic effect was assessed with light microscopy 4 days after infection in T47D and MDA-MB-231 cells.

Cells infected with Ad5PB_RSV-NIS at MOI 1 were monitored for viral-induced cytopathic effect (CPE) by light microscopy. Four days after infection, the morphology of the MDA-MB-231 cells was unchanged; however, clear signs of CPE in T47D are detected under the same conditions of infection (Figure 4).

**Figure 5:**
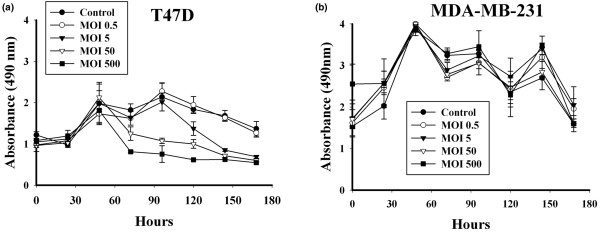
Cell-proliferation assay. **(a) **T47D and **(b) **MDA-MB-231. Cells were infected with Ad5PB_RSV-NIS, and cell viability was monitored daily by using MTS assay.

To address the issue of virus specific cell killing, the permissive cell line T47D was infected with the Ad5AMUCH_RSV-NIS CRAd at increasing MOIs and then assayed daily by MTS. The results were compared with those obtained with the nonpermissive cell line MDA-MB-231. The T47D cells showed a reduced viability that was both time and dose dependent (Figure 5a). Conversely, MDA-MB-231 viability was completely refractory to virus-mediated cell killing (Figure 5b).

**Figure 6:**
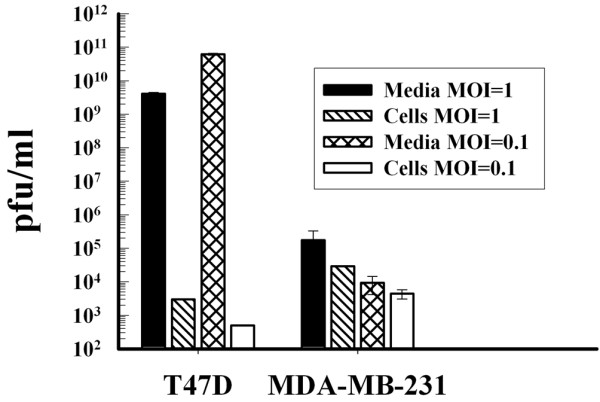
Cell-specific replication of Ad5AMUCH_RSV-NIS. T47D and MDA-MB-231 cells were infected with Ad5AMUCH_RSV-NIS at multiplicity of infection (MOI) 1 or MOI 0.1. Viral progeny were isolated from media and cells and quantitated with plaque assay on 293 cells.

Virus-progeny production after infection with the Ad5AMUCH_RSV-NIS CRAd was measured in T47D, and MDA-MB-231 cells 3 days after infection at MOI 1 or 0.1 by burst assay (Figure 6). To distinguish between released and intracellular virus, media and cell were separated, and virus was purified separately from each. The amount of virus produced by the T47D cells was 6 orders of magnitude greater than the amount produced by the MDA-MB-231 cells.

Taken together, these data demonstrate that replication of the Ad5AMUCH_RSV-NIS virus is stringently restricted to MUC-1-positive breast cancer cells, defining this virus as a conditionally-replicating adenovirus or CRAd.

### NIS expression and radioiodine uptake

**Figure 7:**
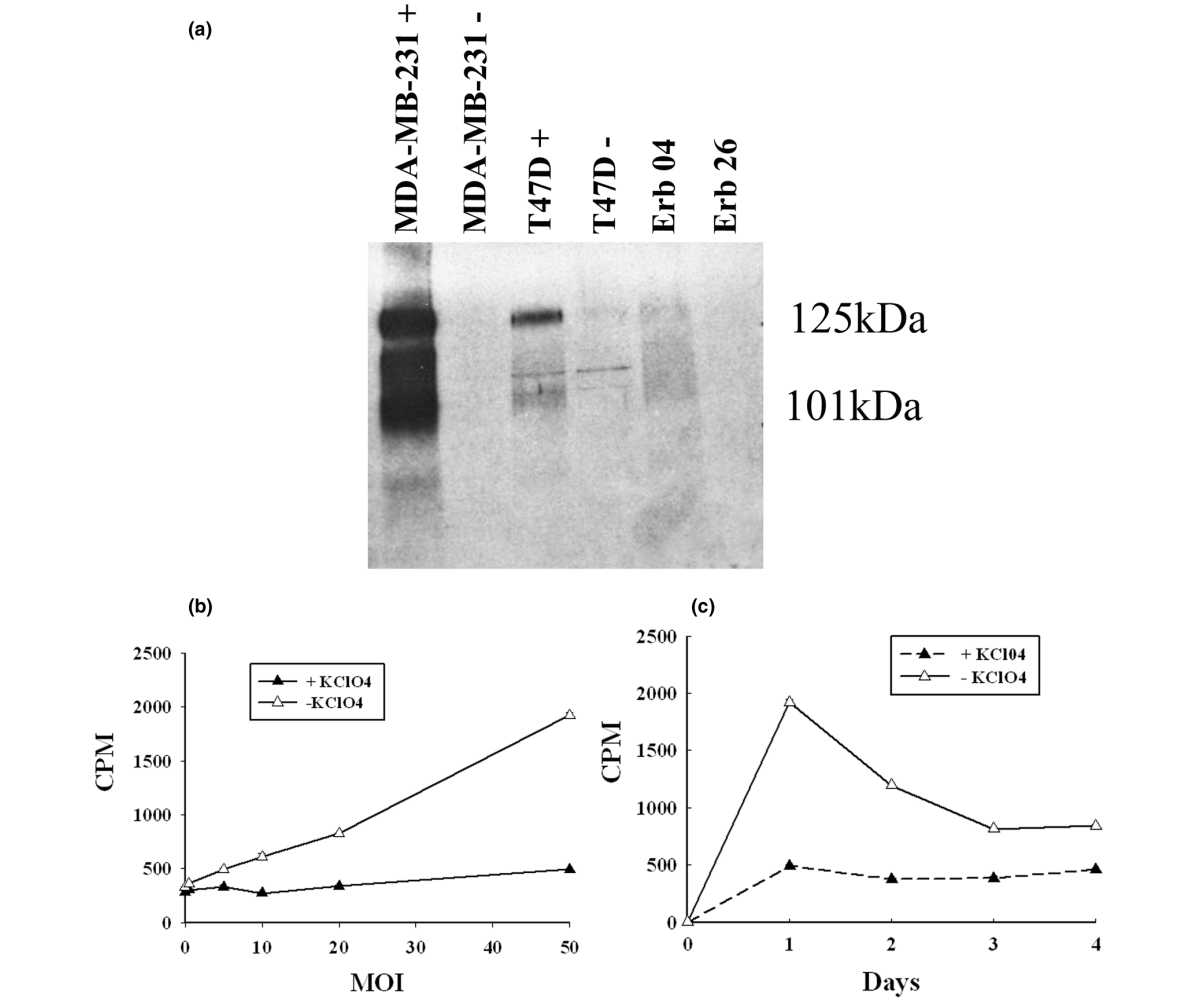
Analysis of sodium iodide symporter (NIS) expression. **(a) **NIS expression assayed with Western blotting in Ad5AMUCH_RSV-NIS-infected cells. Membrane proteins were prepared 24 hours after infection. **(b) **Radioiodine uptake dose-response. T47D cells were infected at the indicated multiplicity of infection (MOI). Radioiodine uptake was measured 24 hours after infection. **(c) **Kinetics of NIS-mediated radioiodine uptake. T47D cells were infected at MOI 20 with the Ad5AMUCH_RSV-NIS conditionally replicating adenovirus (CRAd), and ^125^I uptake was measured daily.

Expression of the NIS protein was examined with Western blotting by using a mouse monoclonal anti-human NIS antibody [30]. Figure 7a shows that T47D and MDA-MB-231 cells transfected with the Ad5PB_RSV-NIS CRAd expressed NIS, which was detected as a broad band spanning from 130 Kd to 110 kDa. Visualization of the NIS band as a smear is expected; it reflects differences in NIS glycosylation [30,31]. Expression of the NIS gene in the nonpermissive cell line MDA-MB-231 is supported by the RSV ubiquitous promoter and shows that this cell line is susceptible to Ad5 infection.

Having shown that infected cells are capable of expressing membrane-bound NIS, we next investigated whether the protein was functional. Dose-response and kinetic curves of iodine uptake were constructed. Infection of T47D cells with Ad5AMUCH_RSV-NIS at MOI 20 showed maximal uptake at 24 hours after infection, followed by a twofold decrease in ^125^I uptake at later time points (Figure 7b). Radioiodine uptake was inhibited by KClO_4_, a well-known inhibitor of NIS activity [3]. We infer from this result that an optimal time window exists for NIS-mediated radioiodine uptake in the permissive cells, after which, uptake, and as a consequence, NIS expression declines. This might be due to virus replication and induction of CPE. Twenty-four hours after infection, a radioiodine uptake dose-response to increasing MOI concentrations was built. Figure 7c shows that radioiodine uptake was linear over the range of MOI used in the experiment.

**Figure 8:**
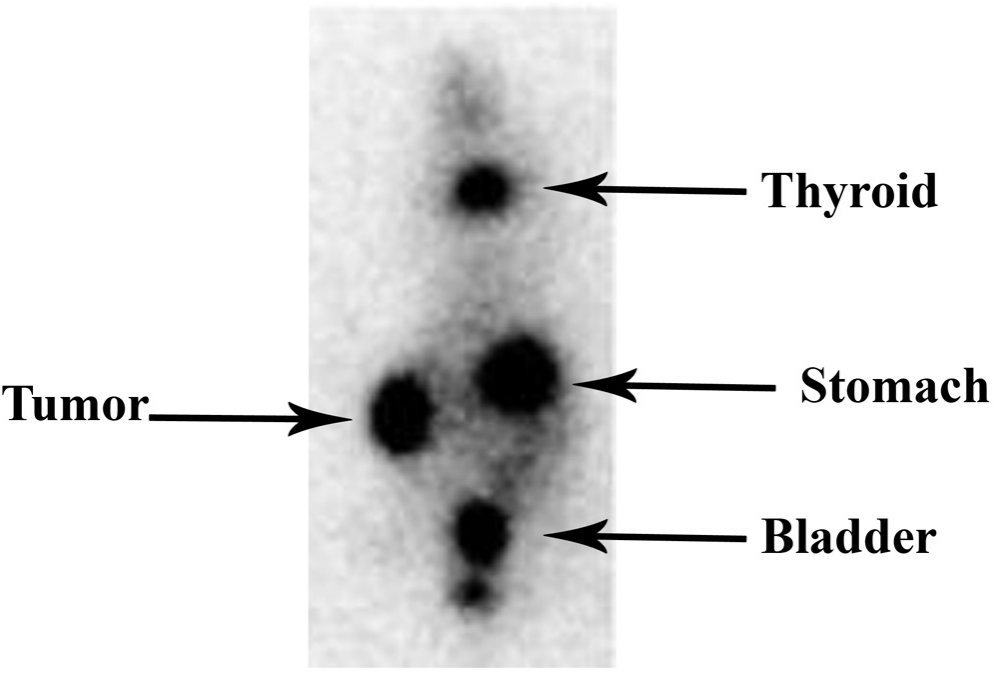
Radioiodine imaging of T47D xenografted tumors. T47D xenografted tumors were established in athymic nude mice. The left-flank tumor was injected with 10^11 ^vp of the Ad5AMUCH_RSV-NIS CRAd, and 48 hours later, 0.5 mCi of ^123^I was administered intraperitonially. Images were captured using a γ camera 1 hour after radioisotope administration.

Having shown in cell culture that Ad5AMUCH_RSV-NIS can infect, multiply, and direct NIS expression in permissive cells, we wished to confirm that viral infection and NIS expression also take place *in vivo*. Figure 8 reveals strong ^123^I uptake by the T47D tumor that has been injected with the Ad5PB_RSV-NIS CRAd 48 hours after infection. Quantitation of the image yielded an uptake in infected tumors of more than 30% of the injected dose at 1 hour. The stomach and thyroid also were imaged because of native NIS expression, and the bladder, because of tracer excretion [9].

Taken together our results demonstrate that Ad5AMUCH_RSV-NIS directs NIS expression in permissive cells before the onset of cell lysis.

## Discussion

Although a number of gene-therapy approaches have been developed for many cancers, clinical trials completed to date have fallen short of expectations [32-36], largely due to limited tumor transduction, and this limitation has been defined as the most important fundamental barrier to effective cancer gene therapy [37]. Our approach to addressing the problem of low tumor transduction is to allow vector replication by using conditionally replicating adenovirus.

We used a transcriptional-regulation method placing the *E1A *gene under control of the tumor-specific promoter MUC-1. The rationale for this choice is based on the observation that transcriptional-dependent regulation seems to be the most efficient strategy for CRAd development [38]. In addition, we inserted the *NIS *gene under control of the powerful but nonspecific RSV promoter. We used a truncated version of the MUC-1 promoter that was previously shown to be specifically active in MUC-1-positive breast cancer cell lines [17]. The rationale and utility for use of this promoter is that more than 90% of breast tumors overexpress the MUC-1 gene [13]. By this method, viral replication, and consequently extended *NIS *expression, will target a wide spectrum of MUC-1-positive breast tumors.

In the present study, we confirmed that this MUC-1 promoter version restricts viral protein synthesis, cytopathic effect, cell lysis, and infectious viral progeny production to MUC-1-positive breast cancer cells. Moreover, the bulk of viral progeny produced by the MUC-1-positive breast cancer cell line T47D was found in the media, indicating that viral infection of T47D cells with the Ad5AMUCH_RSV-NIS CRAd resulted in cell lysis and subsequent release of infectious viral particles. We hypothesize that the release of infectious viral particles should allow wide viral spread within tumors. Virus specificity was demonstrated by using the MUC-1-negative breast cancer cell line MDA-MB-231. The MDA-MB-231 cell line expresses cell-surface CAR receptor because we visualized CAR expression in membrane-bound electrophoresed proteins. It supports limited E1A and hexon expression and supports NIS expression. All these data confirm that MDA-MB-231 is infected by the Ad5AMUCH_RSV-NIS, yet it is resistant to CRAd-induced oncolysis or viral replication or both. These data demonstrate that the Ad5AMUCH_RSV-NIS CRAd is stringently dependent on MUC-1 overexpression and should not replicate within normal tissue, where MUC1 expression is minimal.

The NIS protein must be properly targeted to the cell plasma membrane to be functional [39]. Our results also showed that the Ad5AMUC_RSV-NIS CRAd directs correct NIS expression and thus radioiodine uptake *in vitro *and *in vivo*. Under the control of the RSV promoter, the NIS protein was correctly targeted to the cell plasmamembrane, thereby promoting high levels of iodine uptake in T47D cells. Our results also show that in MUC-1-negative CAR-positive breast cancer tumors, the NIS gene also can be expressed, because it is under the control of the ubiquitous RSV promoter. Here, the cell line MDA-MB-231 showed very strong NIS protein expression by Western blot, which translated to robust radioiodine uptake (data not shown). Hence the Ad5AMUCH_RSV-NIS CRAd could be used as a non-replicating NIS-expressing vector.

However, in this case, NIS gene expression was transient, thus narrowing the window for radioiodine therapy and loss of the combined radiovirotherapy.

A discrepancy is apparent in the kinetics of NIS expression and luciferase expression that can be explained by several influencing factors; First, the *luciferase *gene was cloned at the E1 region of a nonreplicating adenovirus, under the control of the MUC-1 promoter, whereas the *NIS *gene was cloned at the E3 region of a replicating adenovirus under the RSV promoter. Second, the stability of two very different mRNAs and proteins cannot be assumed to be equal.

Finally, NIS is an integral membrane protein, whereas luciferase remains cytoplasmic. Therefore, the pattern of expression of luciferase and NIS under these conditions is not expected to overlap precisely.

*NIS *is already a therapeutic gene, in that its native expression in thyroid cells is used for therapy for thyroid cancer and hyperthyroidism, where its efficacy is very high [6]. *NIS *expression could represent an ideal therapeutic gene for breast cancer therapy, because it allows a large bystander effect. Because of the physics of ^131^I decay, which occurs primarily through emission of a β particle that traverses up to 2 mm within the tissue. Thus, not every cell must be transfected and express NIS to be affected by radioiodine therapy. This is critical to the effectiveness of any cancer gene-therapy strategy because of the difficulty in achieving 100% transfection with the therapeutic gene, even in the case of a replicating virus. Moreover, NIS expression can be directly monitored and quantitated noninvasively. The uptake of radioiodine in tissues can be quantitated, and both the distribution and the quantity of NIS expression can be easily and safely monitored noninvasively.

The limited efficacy observed in clinical trials of cancer gene therapies to date has suggested that combinatorial therapies to treat cancer will likely be more effective and possibly required for complete tumor eradication. The hypothesis developed from this observation is that attacking tumor cells through different mechanisms of action may prevent tumor cells from developing resistance to the treatment combination and may more effectively induce immunity against the tumor [40]. We hypothesize that, because Ad5AMUC_RSV-NIS CRAd harbors the *NIS *gene, it should improve the therapeutic value of the CRAd by allowing multimodal therapy in a single agent combining viral-mediated tumor lysis as well as radioiodine-mediated therapy.

## Conclusions

In conclusion, the features of Ad5PB_RSV-NIS CRAd address favorably the major hurdles identified in gene-therapy clinical trials for anticancer treatments. Further evaluation, including efficacy studies in animal models, is needed to determine the potential of this radio/virotherapy approach to affect breast cancer treatment.

## Abbreviations

Ad5: adenovirus serotype 5; bGH: bovine growth hormone; BIS-TRIS: bis(2-hydroxyethyl)iminotris(hydroxymethyl)methane; CAR: coxsackievirus adenovirus receptor; CIP: calf intestine phosphatase; CPE: cytopathic effect; CRAd: conditionally replicating adenovirus; EDTA: ethylenediaminetetraacetic acid; HBSS: Hanks' buffered salt solution; LTR: long terminal repeat; MOI: multiplicity of infection; MTS: [3-(4,5-dimethylthiazol-2-yl)-5-(3-carboxymethoxyphenyl)-2-(4-sulfophenyl)-2H-tetrazolium; MUC-1: mucin-1; NIS: sodium iodide symporter; PBS: phosphate-buffered saline; PCR: polymerase chain reaction; polyA: polyadenylation signal; RSV: Rous sarcoma virus; RT: room temperature; TBS-T: Tris-buffered saline Tween-20.

## Competing interests

The authors declare that they have no competing interests.

## Authors' contributions

MAT constructed the CRAd, participated in the design of the study, performed FACS analysis, luc assay, and cytopathic effect, supervised the work of MJO and ER, and drafted the manuscript. MJO performed MTS and cell-specific replication. JD constructed, amplified, and purified AdEasyGL3B-Muc with MY. EB performed Western blots and radioiodine uptake. MY constructed, amplified, and purified AdEasyGL3B-Muc AdEasyGL3B-Muc with JD. JCM conceived of the study, participated in its design and coordination, and helped to draft the manuscript.
